# Abundance of clinical variants in exons included in multiple transcripts

**DOI:** 10.1186/s40246-018-0166-2

**Published:** 2018-06-28

**Authors:** Sankar Subramanian

**Affiliations:** GeneCology Research Centre, The University of the Sunshine Coast, 90 Sippy Downs Drive, Sippy Downs, Qld 4556 Australia

**Keywords:** Constitutive exons, Alternatively spliced exons, Rate of protein evolution, Pathogenic variants

## Abstract

Previous studies showed that the magnitude of selection pressure in constitutive exons is higher than that in alternatively spliced exons. The intensity of selection was also shown to be depended on the inclusion level of exons: the number of transcripts that include an exon. Here, we examined how the difference in selection pressure influences the patterns of clinical variants in human exons. Our analysis revealed a positive relationship between exon inclusion level and the abundance of pathogenic variants. The proportion of pathogenic variants in the exons that are included in > 10 transcripts was 6.8 times higher than those in the exons included in only one transcript. This suggests that the mutations occurring in the exons included in multiple transcripts are more deleterious than those present in the exons included in one transcript. The findings of this study highlight that the exon inclusion level could be used to predict the mutations associated with diseases.

## Background

One of the major tasks in clinical genomics is the identification of mutations associated with human genetic diseases. Typically, genome-wide genetic studies identify a large number of variants that have potential association with a disease [1]. However, to narrow this search and to pinpoint the variants that most likely cause a disease, a number of methods have been developed [2–7]. These methods use the evolutionary conservation of nucleotide positions and/or the functional consequences of mutations to distinguish disease-associated variants from neutral and benign variants. Some of these methods integrate a large number of functional annotations to identify deleterious mutations [2, 5], and hence, any new annotation will enhance the predictability of disease-associated mutations.

Previous studies showed that the rate of protein evolution in alternatively spliced exons was much higher than that observed in constitutive exons [8, 9]. Furthermore, the inclusion level of exons (the number or proportion of transcripts that include an exon) was found to be modulating the rate of evolution of proteins in mammals [8, 9]. These findings suggest that the magnitude of selection constraints is higher in exons included in multiple transcripts than that in exons included in a single transcript. Furthermore, a previous study showed that brain-expressed exons under purifying selection have more de novo mutations [10]. However, how the variation in selection intensity between different exons influence the distribution of deleterious and diseases-associated mutations is unclear. Hence, to investigate this, we obtained the alignment of the protein-coding genes for the human-macaque pair, population polymorphisms, and clinical pathogenic variations in human exomes. Using this large-scale data, we examined the relationship between exon inclusion levels and the fraction of deleterious and pathogenic variants.

## Methods

Whole genome pairwise alignment of the human-macaque pair (hg38.rheMac3.net.axt - Dec. 2013) was downloaded from the UCSC genome data repository (https://genome.ucsc.edu/). Using the reference gene annotations, pairwise exonic alignments were extracted and the number of nonsynonymous and synonymous substitutions as well as sites were estimated for each exon. Nonsynonymous and synonymous substitutions observed for individual exons were summed separately for each class of exons, and Kimura two parameter correction [11] was applied to estimate evolutionary distance at nonsynonymous and synonymous sites. We used the analytical variance to calculate the standard error [11].

The number of exons in each transcript, intron-exon boundaries and the number of transcript in each protein coding gene (Ensembl genes 92 – GRCh38.p12) were obtained from the *Ensembl* genome resource (www.ensembl.org). Exons were then grouped into 10 classes based on the number of transcripts that included them (1–10 transcripts), and the 11th class consists of the exons included in > 10 transcripts (exon inclusion level). We have also grouped exons into 10 categories based on the proportion of alternative transcripts that included them (exon inclusion ratio). Since our analysis is based on deleterious nonsynonymous SNVs, only the coding exons were included for the analysis.

Human variation data along with the annotations of functional consequences (Ensembl release 92 – GRCh38) was also downloaded from the *Ensembl* genome resource (https://www.ensembl.org/), and synonymous and nonsynonymous variations were extracted. Deleterious variations were determined based on the method, *PolyPhen2*, and only the variants designated as “probably deleterious” were included in the analysis [12]. Based on the annotations of the *ClinVar* database, variants that were denoted as “pathogenic” were used for further analysis [13]. Our final dataset included 5.1 million nonsynonymous variations of which 1.7 million were predicted to be deleterious by *PolyPhen2* and 25,883 were determined to be pathogenic by the *ClinVar* database. The proportion of deleterious nonsynonymous variants was calculated as the number of “probably deleterious” variations divided by the total number of nonsynonymous variations. Similar calculation was followed to obtain the proportion of pathogenic nonsynonymous variations, and the binomial variance was used to estimate the standard error. To examine the strength of correlations, the nonparametric Spearman’s rank correlation was used.

## Results and discussion

**Fig. 1:**
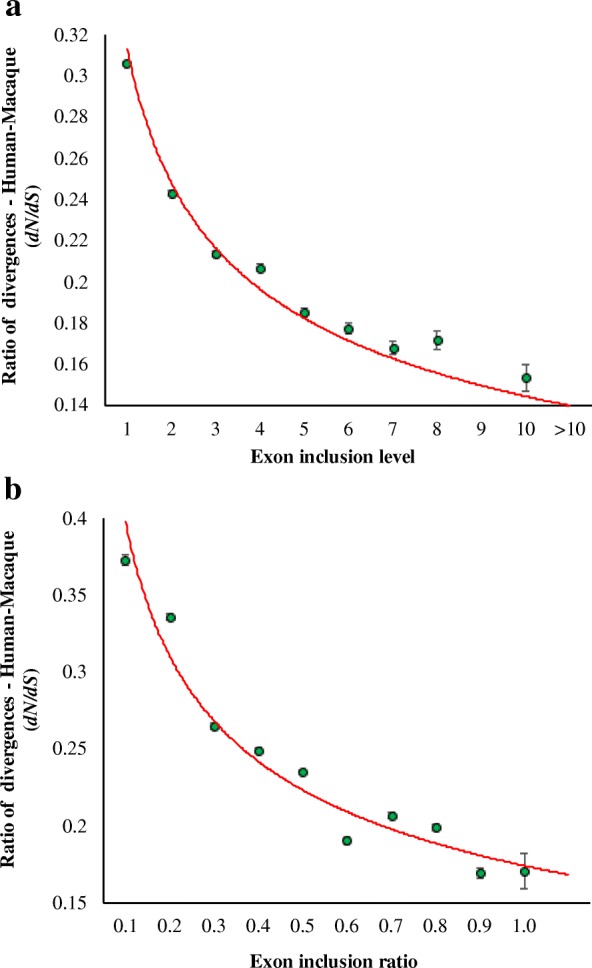
Relationship between the exon inclusion measures and the ratio of nonsynonymous-to-synonymous divergences (*dN/dS*). **a** Exon inclusion level. **b** Exon inclusion ratio. Synonymous and nonsynonymous divergences were estimated using the Kimura two parameter model [11]. Error bars show the standard error of the mean, which were calculated using the analytical variance of the Kimura two parameter method [11]. The relationships were highly significant (*ρ* = − 0.98 and *ρ* = − 0.95 respectively; *P* < 0.001) using the Spearman’s rank correlation. Note that in **a**, the last value (> 10 exons) on the *X*-axis was taken as 11 exons to calculate the correlation coefficient and significance

To examine the effects of selection intensity on human exons, we estimated the divergence at nonsynonymous (*dN*) and synonymous (*dS*) positions of the protein-coding genes of the human-macaque comparison. We then plotted the ratio of divergences (*ω* = *dN*/*dS*) against the number of transcripts that included the exon (exon inclusion level) (Fig. 1). This revealed a highly significant negative correlation between the exon inclusion level and *dN*/*dS* ratio (*P* < 0.001) (Fig. 1a). The *dN/dS* estimated for exons included in > 10 transcripts was 2.4 times smaller than that estimated for exons included in only one transcript. We also used another measure—exon inclusion ratio, which is the proportion of alternative transcripts that included an exon. Figure 1b shows that exon inclusion ratio also has a significant negative relationship with *dN/dS*. The *dN/dS* observed for exons included in > 90% of the transcripts was 2.2 times smaller than that estimated for exons included in 10% of the transcripts. The above results suggest that protein evolution was under high selection pressure for the exons included in many transcripts.

**Fig. 2:**
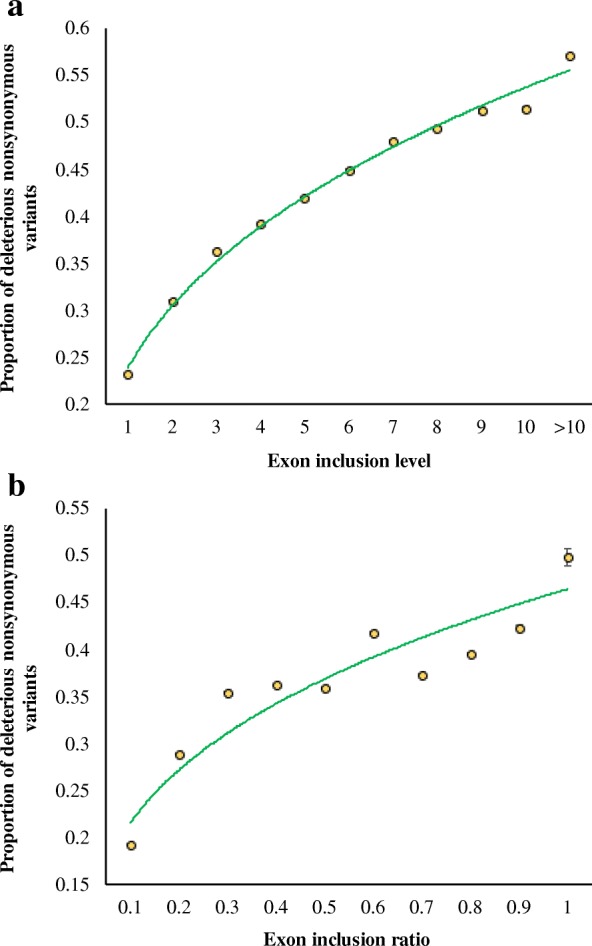
Correlation between exon inclusion measures and the proportions of deleterious variants. **a** Exon inclusion level. **b** Exon inclusion ratio. Deleterious variants predicted by *PolyPhen2*. Error bars are the standard error of the mean, which were calculated using the binomial variance. The relationships were highly significant (*ρ* = 1; *P* < 0.001)

The higher selection pressure on exons included in many transcripts suggests the relative importance of these exons compared to those included in one or a few transcripts. Hence, any mutation in the former will be more deleterious than that in the latter. This was tested by examining the proportion of deleterious nonsynonymous variants: variants that were predicted by the method *PolyPhen2* based on sequence conservation levels and a number of functional annotations. Our results clearly showed a high significant positive correlation (*P* < 0.001) between the exon inclusion levels and the proportion of deleterious nonsynonymous variations (Fig. 2a). About 23% of the nonsynonymous variations observed in the exons included in one transcript were found to be deleterious in nature. However, this fraction was 57% for the exons included in > 10 transcripts, which is 2.5 times higher that observed for the exons included in only one transcript. A similar relationship (*P* < 0.001) was observed when exon inclusion ratio was used (Fig. 2b), and the magnitude of difference in the proportion of deleterious SNVs between the two extreme classes was 2.6 times (19% vs 50%). These population polymorphism-based results further confirm those obtained based on inter-species comparison.

**Fig. 3:**
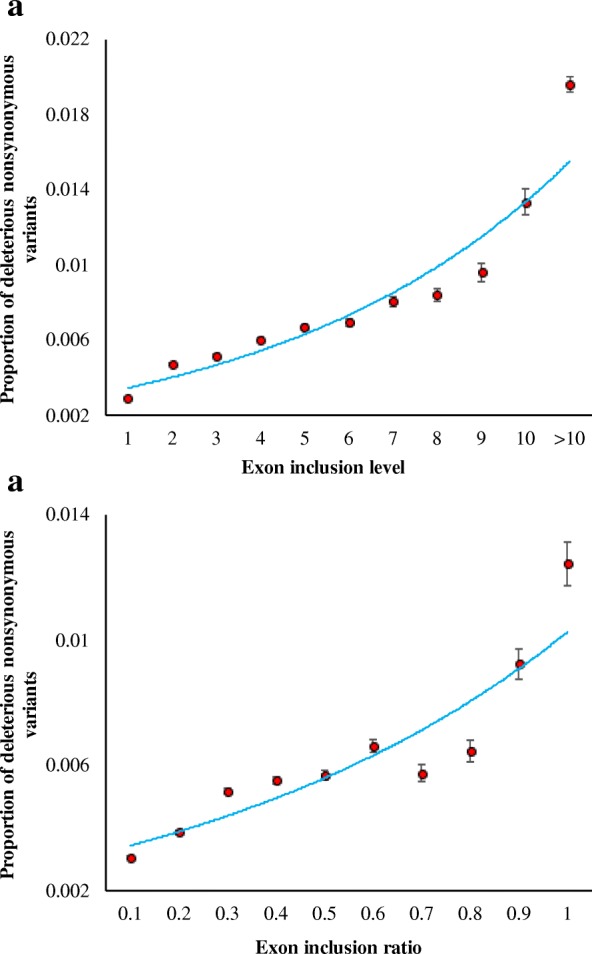
Correlation between exon inclusion measures and the proportions of pathogenic variants. **a** Exon inclusion level. **b** Exon inclusion ratio. Clinical pathogenic variants were identified using the *ClinVar* database. Error bars are the standard error of the mean, which were calculated using the binomial variance. The relationships were highly significant (*ρ* = 0.95 and *ρ* = 0.96 respectively; *P* < 0.001)

We then examined the above relationship for pathogenic variations: variants in the *ClinVar* database that were well-known to be causing or associated with a genetic disease. Our results showed a similar positive correlation (*P* < 0.001) between the exon inclusion level and the fraction of pathogenic variations (Fig. 3a). However, the magnitude of this relationship was much larger than that observed for the predicted deleterious variants. The proportion of pathogenic nonsynonymous variations in exons included in > 10 transcripts was 1.96%, which is 6.8 times higher than that observed for the exons included in one transcript (0.29%). We performed similar analysis using exon inclusion ratio, which also produced a highly significant positive relationship (*P* < 0.001). We observed a fourfold difference (1.24 vs 0.31%) in the fraction of pathogenic amino acid variations between the two extreme exon inclusion ratio categories (Fig. 3b).

We also investigated whether the patterns observed above hold true for genes evolving under different selection pressures. For this purpose, we separated genes based on the level of selection constraints measured by the *dN/dS* ratio and grouped them into three classes. We then compared the exon inclusion level and proportion of deleterious SNVs in the three gene classes (Table 1). In the case of highly constrained genes (*dN/dS* < 0.25), the proportion of deleterious SNVs in the exons included in > 5 transcripts was 2.1 times higher than that in the exons included in only one transcript (0.515 vs 0.247). A similar difference of 2.2 times (0.430 vs 0.197) was observed for the less constrained genes (*dN/dS* > 0.5) as well. Similar results were observed comparing the values based on the exon inclusion ratio (Table 1). The proportions of clinical variants in genes under varying selection pressure were also found to be comparable. For instance, the analysis of genes under high selection pressure (*dN/dS* < 0.25) showed that the fraction of pathogenic variants in the exons included in > 5 transcripts was 3.4 times higher than that estimated for the exons included in only one transcript (0.0116 vs 0.0034). In the case of less constrained genes (*dN/dS* > 0.5), this difference was 3.5 times (0.0056 vs 0.0016), which is very similar to the former. A very similar pattern was also observed for the results based on the exon inclusion ratio. These results clearly demonstrate that the patterns reported in this study are very similar across genes under varying levels of selection pressure.

**Table 1:** Proportion of deleterious SNVs and clinical variants in genes evolving under varying levels of selection intensity

Selection intensity dN/dS	Exon inclusion level (number of transcripts in which the exon is included)			Exon inclusion ratio (proportion of transcripts in which the exon is included)		
	1	2–5	> 5	0.1	0.2–0.5	> 0.5
Deleterious SNVs						
< 0.25	0.2466 (0.0006)	0.3748 (0.0004)	0.5155 (0.0010)	0.1598 (0.0028)	0.2785 (0.0010)	0.3417 (0.0019)
0.25–0.5	0.2243 (0.0008)	0.3391 (0.0006)	0.4538 (0.0015)	0.1956 (0.0015)	0.3248 (0.0006)	0.3728 (0.0012)
> 0.5	0.1972 (0.0012)	0.2994 (0.0010)	0.4301 (0.0026)	0.2027 (0.0010)	0.3666 (0.0004)	0.4257 (0.0008)
Clinical variants						
< 0.25	0.0034 (0.0001)	0.0062 (0.0001)	0.0116 (0.0002)	0.0015 (0.0003)	0.0027 (0.0001)	0.0036 (0.0002)
0.25–0.5	0.0025 (0.0001)	0.0041 (0.0001)	0.0065 (0.0002)	0.0018 (0.0002)	0.0041 (0.0001)	0.0044 (0.0002)
> 0.5	0.0016 (0.0001)	0.0028 (0.0001)	0.0056 (0.0004)	0.0034 (0.0001)	0.0059 (0.0001)	0.0079 (0.0002)

## Conclusions

The results of the present study highlight the abundance of clinical mutations associated with diseases in exons that are included in multiple transcripts. The excess of harmful mutations in these exons was attributed to the high magnitude of selective constraints on them [3, 14]. Because an exon required for many transcripts is more vital to an organism than that is needed for a single transcript. Hence, mutations in the former will have more severe deleterious effects than those in the latter. These findings could be useful in identifying pathogenic mutations, and exon inclusion level could be used as an indicator measure to detect deleterious variants.

## Abbreviations

*dN*: Divergence at nonsynonymous sites
*dN/dS*: Ratio of divergences at nonsynonymous-to-synonymous sites
*dS*: Divergence at synonymous sites
